# Quantitation of the Rank-Rankl Axis in Primary Biliary Cholangitis

**DOI:** 10.1371/journal.pone.0159612

**Published:** 2016-09-15

**Authors:** Ana Lleo, Zhaolian Bian, Haiyan Zhang, Qi Miao, Fang Yang, Yanshen Peng, Xiaoyu Chen, Ruqi Tang, Qixia Wang, Dekai Qiu, Jingyuan Fang, Cristina Sobacchi, Anna Villa, Luca Di Tommaso, Massimo Roncalli, M. Eric Gershwin, Xiong Ma, Pietro Invernizzi

**Affiliations:** 1 Liver Unit and Center for Autoimmune Liver Diseases, Humanitas Clinical and Research Center, Rozzano, Milan, Italy; 2 State Key Laboratory for Oncogenes and Related Genes, Key Laboratory of Gastroenterology & Hepatology, Ministry of Health, Division of Gastroenterology and Hepatology, Ren Ji Hospital, School of Medicine, Shanghai Jiao Tong University, Shanghai Cancer Institute, Shanghai Institute of Digestive Disease, Shanghai, China; 3 Istituto di Ricerca Genetica e Biomedica, Consiglio Nazionale delle Ricerche, Milano Italy; 4 Humanitas Clinical and Research Center, Rozzano, Milan, Italy; 5 Pathology Unit, Humanitas Clinical and Research Center, Rozzano, Milan, Italy; 6 Department of Biomedical Sciences, Humanitas University, Rozzano, Milan, Italy; 7 Division of Rheumatology, Allergy, and Clinical Immunology, University of California at Davis School of Medicine, Davis, CA, United States of America; 8 International Center for Digestive Health, Department of Medicine and Surgery, University of Milan-Bicocca, Milan, Italy; Karolinska Institutet, SWEDEN

## Abstract

There is substantial data that suggests an abnormality of innate immunity in patients with primary biliary cholangitis (PBC) which includes the transcription factor nuclear factor-kB (NF-kB) and well as downstream inflammatory signaling pathways. In addition, ImmunoChip analysis has identified a novel PBC-associated locus near the receptor activator of NF-kB ligand (RANKL) gene. Based on these observations, we investigated the role of the RANKL axis in the liver of patients with PBC compared to controls. We used immunohistochemistry to quantitate liver expression of RANKL, its receptor (RANK), and importantly the decoy receptor osteoprotegerin (OPG), including a total of 122 liver samples (PBC = 37, primary sclerosing cholangitis = 20, autoimmune hepatitis = 26, chronic hepatitis B = 32 and unaffected controls = 7). In addition, we studied RANKL-RANK-OPG co-localization in CD4 and CD8 T cells, B cells, dendritic cells, macrophages, NK, NKT cells, hepatocytes, and cholangiocytes. We report herein that RANK is constitutively expressed by cholangiocytes in both unaffected and diseased liver. However, cholangiocytes from PBC express significantly higher levers of RANK than either the unaffected controls or liver diseased controls. CD4, CD8 and CD19 cells with in the portal areas around bile ducts in PBC express significantly higher levels of RANKL compared to controls. Importantly, the overall hepatic RANKL level and the ratio of hepatic RANKL/OPG correlated with disease severity in PBC. In conclusion, our data indicate a role of RANK-RANKL axis in the innate immune activation in PBC and we hypothesize that the damaged cholangiocytes, which express high levels of RANK, lead to the recruitment of RANKL positive cells and ultimately the classic portal tract infiltrates.

## Introduction

Primary biliary cholangitis (PBC) [1], is a chronic liver disease characterized by progressive destruction of intrahepatic bile ducts resulting in cholestasis, portal tract inflammation, and fibrosis that may progress to cirrhosis and ultimately end-stage liver disease [2, 3]. Although the exact etiology of PBC remains unknown, it is widely recognized that the development of PBC requires one or more environmental factors that initiate an autoimmune response in genetically predisposed individuals [4–6]. Previous work from our group has identified a novel disease-associated locus near the TNFSF11 gene, upstream of the gene encoding the receptor activator of nuclear factor κB (NF-κB) ligand (RANKL) [7]. Even though it is inherently difficult for genome wide association studies to identify a specific gene, a large amount of data suggest that RANKL may play a relevant role in PBC.

Since the discovery of the receptor activator of NF-κB (RANK) in the late 1990s, the RANKL/RANK/osteoprotegerin (OPG) system has been implicated in regulating immune responses; RANK, also known as TRANCE Receptor, is a type І membrane protein that is expressed on the surface of osteoclasts, dendritic cells and mammary gland epithelial cells physiology [8–11]. The cognate ligand for RANK, RANKL, is a member of the tumor necrosis factor (TNF) cytokine family, which functions as a key factor for osteoclast differentiation and activation. In addition, the RANKL/RANK pathway serves a critical role in the immune system and augments the ability of dendritic cells (DC) to stimulate naive T cell proliferation and enhance DC survival [12]. It also has important regulatory functions in lymph node organogenesis and lymphocyte differentiation [13]. OPG is a decoy receptor for RANKL. Through the binding of RANKL, OPG inhibits NF-kB, a key regulator of inflammation, innate immunity and immune cell survival and differentiation [9, 14].

The essential function of the RANKL-RANK-OPG axis in immune system is relevant to autoimmunity. The gene encoding RANKL, or TNFSF11, is positioned within a confirmed loci implicated in Crohn’s disease [15, 16]. Moreover, elevated serum levels of OPG and soluble RANKL have also been reported in rheumatoid arthritis (RA) [17, 18]. Interestingly, RANKL mRNA is present in the synovial lining layer in RA but not in normal synovia [19]. The role of OPG and RANKL in bone remodeling and development of osteoporosis in PBC has been previously investigated [20]. We report herein data that strongly suggest abnormal RANK-RANKL signaling in PBC; further investigation is needed to better understand the recruitment of the inflammatory infiltrate that targets biliary cells.

## Materials and Methods

### Human Subjects

A total of 115 patients with chronic liver diseases and 7 unaffected controls were enrolled in this study. These patients included 37 subjects with well characterized PBC, 26 subjects with autoimmune hepatitis (AIH), 32 subjects with chronic hepatitis B (CHB), and 20 subjects with primary sclerosing cholangitis (PSC). Based on Scheuer’s classification, 37 PBC patients were staged, including stage I and II (n = 14) and stage III and IV (n = 23) [21]. The clinical data are summarized in **Table 1** and the diagnosis was based on established criteria for PBC [22], PSC [23], AIH [24], and CHB [25], respectively. Unaffected subjects were included as controls (HC); these samples were derived from patients undergoing liver surgery for benign lesions (haemangioma resection, n = 3) and liver donors for Orthotropic Liver Transplantation (n = 4). None of these individuals had evidence of liver pathology. Samples from PBC subjects as well as AIH, CHB, and HC were collected in China, whereas PSC samples were obtained from subjects followed at the Center for Autoimmune Liver Diseases, Humanitas Clinical and Research Center.

Informed consent in writing was obtained from all subjects and the study protocol was approved by the Ethics Committee of Renji Hospital, Shanghai Jiao Tong University and Humanitas Clinical and Research Center, Rozzano, Milan, Italy.

**Table 1:** Clinical data of PBC, AIH, CHB, PSC and unaffected controls (CTR). Mean values ± standard deviation unless otherwise stated.

		PBC (n = 37)	AIH (n = 26)	CHB (n = 32)	PSC (n = 20)	CTR (n = 7)
Female/Male		31/37	21/26	11/32	8/20	4/7
Age (years)		47±12	44±10	42±11	44±16	43±2
ALT (UI/l)		84±72	186±158	150±16	122±108	26±10
AST (UI/l)		65±73	131±118	102±12	96±84	18 ±4
AKP(UI/l)		243±146	90±28	82±24	358±353	71±17
GGT (UI/l)		290±321	90±52	72±61	365±270	31±11
TBI (μmol/L)		26±15	24±13	23±13	32±36	6±1
AMA (n)		27	n.a	n.a	n.a	n.a.
Fibrosis	F0	0	2	4	7	7
	F1	5	3	5	0	n.a.
	F2	9	8	9	0	n.a.
	F3	14	7	8	9	n.a.
	F4	9	6	6	4	n.a.

### Immunohistochemistry

All liver samples were fixed in 10% formalin and embedded in paraffin before cutting into 4μm sections. Immuohistochemistry staining of RANKL, RANK and OPG was performed with a Leica BondTM system (Leica, Germany). The liver sections were first treated for heat mediated antigen retrieval with sodium citrate buffer (pH6, epitope retrieval solution 1) for 20 minutes, followed by incubation with anti-RANKL antibody(1:100), anti-RANK antibody (1:150) and anti-OPG antibody (1:200) (Abcam, Cambridge, MA) for 20 minutes at room temperature, then developed with a horse radish peroxidase-conjugated compact polymer system. 3, 3′-diaminobenzidine was used as the chromogen. The liver sections were then counterstained with haematoxylin. All sections were visualized using a light microscope (Olympus, Japan) with five fields randomly selected. The numbers of RANKL or OPG positive cells were quantified at high-power field (HPF) (40×10 magnification). The level of RANK expression in each section was scored using a scale of 1 to 4 based on the positive area in each portal tract area: 1, 0–25%; 2, 26–50%; 3, 51–75%; and 4, 76–100%. According to the intensity, the signal level of RANK in the biliary epithelial cells was scored. Weak signal was scored as 1; Mild signal was scored as 2; Strongly positive was scored as 3. Each slide was given an average score based on all of the evaluated portal tracts. A single unbiased "blinded" pathologist analyzed all samples.

Confocal laser scanning microscopy was used to define the data in PBC [26]. Briefly, antigen retrieval was carried out in citrate buffer (pH 6.0) in a microwave for 15 minutes. After cooling, the liver sections were blocked with 10% non-immune goat serum for 15 minutes. The sections were then incubated with two primary antibodies, one of the antibodies against RANKL (1:100), RANK (1:150), or OPG (1:200), and another antibody with CD4 (1:100), CD8 (1:50), CD19 (1:150). The slides were incubated overnight at 4℃. All antibodies were purchased from Abcam except anti-CD8 (Santa Cruz Biotechnology, Santa Cruz, CA) and then incubated with Alexa 488-conjugated donkey anti-mouse IgG and Alexa 594-conjugated donkey anti-rabbit antibody (1:500) (Invitrogen/Life Technologies, UK) for 30 min at room temperature. The nuclei were stained with DAPI (SouthernBiotech, Birmingham, AL). Confocal scanning was performed using an LSM-710 laser-scanning confocal microscope (Carl Zeiss, Jena, Germany).

### Statistical Analysis

All data are reported as mean ± standard error of the mean (SEM). Group means were compared by ANOVA. All the statistical analysis were performed with Prism 5.0 (GraphPad Software La Jolla, CA). A p value of less than 0.05 was considered statistically significant.

## Results

### RANKL/RANK/OPG Are Strongly Expressed in PBC

**Fig 1:**
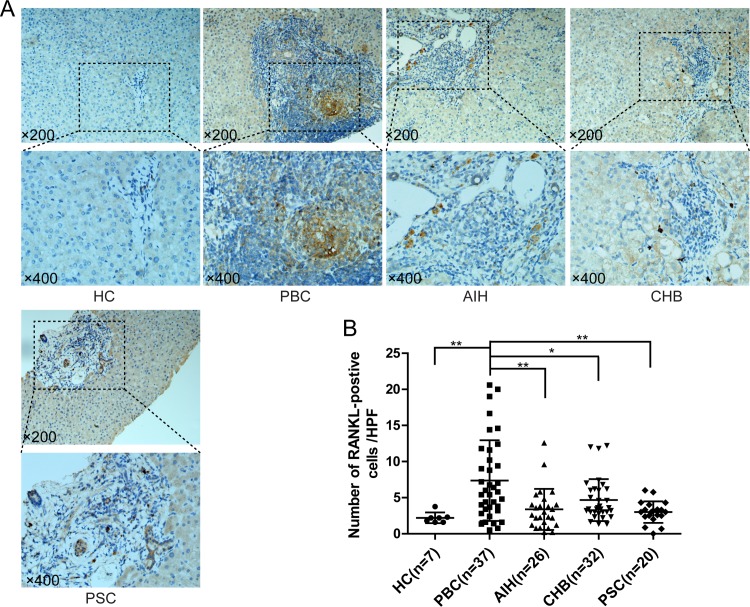
Immunohistochemistry of receptor activator of nuclear factor κB ligand (RANKL) in liver. (A) Representative staining images from PBC, AIH, CHB, PSC and unaffected controls (HC) are shown; (B) Statistical analysis of expressed RANKL in liver. (*, p<0.05; **, p<0.01).

In PBC, RANKL signal was strongly represented in the portal areas around bile ducts (**Fig 1A**), whereas few RANKL-positive cells were found in portal areas of subjects affected by other chronic liver disease, including AIH, CHB and PSC (**Fig 1A**). Moreover, the number of RANKL positive cells was significantly higher in subjects with PBC (7.36±0.92/HPF), compared with unaffected controls (2.21±0.28/HPF) (p<0.01) (**Fig 1A**), as well as in AIH (3.38±0.58/HPF, p<0.01), CHB (4.67±0.51/HPF, p<0.05) and PSC (3.00±0.33/HPF, p<0.01) subjects (**Fig 1B**).

**Fig 2:**
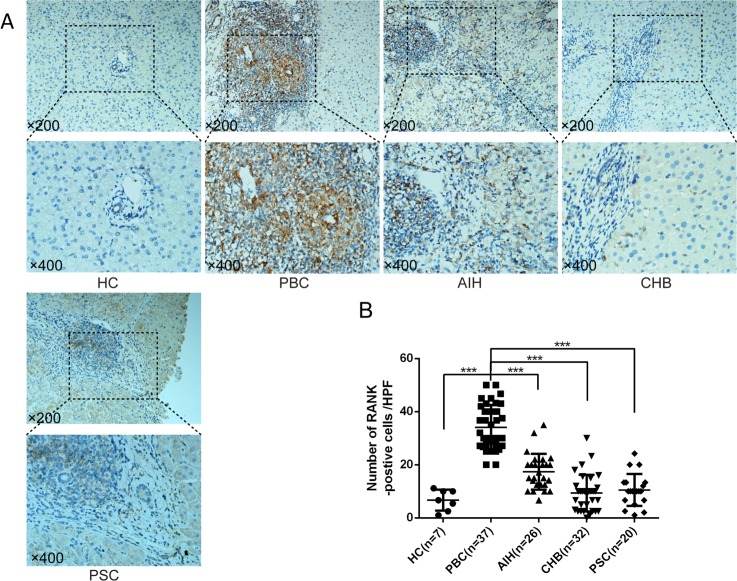
Immunohistochemistry of receptor activator of nuclear factor κB (RANK) in liver. (A) Representative staining images from patients with PBC, AIH, CHB, PSC and unaffected controls (HC) are shown; (B) Statistical analysis of expressed RANKL in liver. (**, p<0.01; ***, p<0.001).

RANK was also studied by immunohistochemistry in subjects affected by chronic liver diseases and unaffected controls (**Fig 2**). RANK was observed in mesenchymal cells in portal areas in PBC; a few RANK-positive cells were located in the hepatic sinusoids (**Fig 2A**). RANK positive cells were significantly higher in chronic liver diseases than unaffected liver controls. Importantly, RANK expression was found significantly higher in PBC (2.41±0.14) than AIH (1.74±0.13, p<0.01), CHB (0.97±0.12, p<0.001), and PSC (1.05±0.13, p<0.001) (**Fig 2B**).

**Fig 3:**
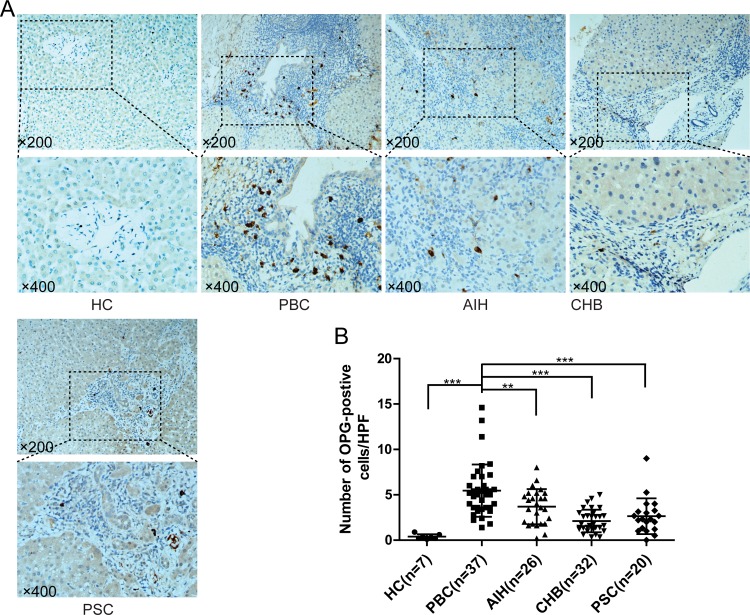
Immunohistochemistry of osteoprotegerin (OPG) in liver. (A) Representative staining images from patients with PBC, AIH, CHB, PSC and unaffected controls (HC) are shown; (B) Statistical analysis of expressed RANKL in liver. (**, p<0.01; ***, p<0.001).

OPG expressing cells were detected in all groups including AIH, PBC, CHB and PSC, as well as unaffected controls. Interestingly, OPG-positive cells were located around the damaged bile ducts in the patients with PBC (**Fig 3A**). OPG expressing cells were significantly higher in PBC (5.45±0.47/HPF), compared with other chronic liver diseases, i.e. AIH (3.89±0.38/HPF, p<0.01), CHB (2.12±0.22/HPF, p<0.001) and PSC (2.81±0.72/HPF, p<0.001) (**Fig 3B**).

### Hepatic RANKL Expression Correlates with Stage of Disease in PBC

**Fig 4:**
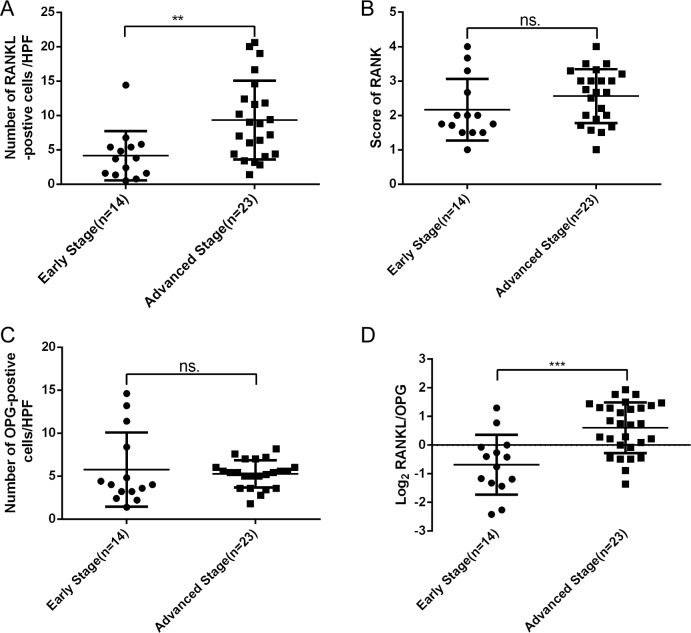
Relationship between RANKL/RANK/OPG with severity in PBC. (A) Elevated hepatic RANKL was correlated with stage in PBC; (B and C) Hepatic RANK and OPG were not elevated with the progress of PBC; (D) The ratio of RANKL/ OPG was higher in late stage PBC compared with early stage. (**, p<0.01; ***, p<0.001; ns., no significance).

Importantly, a significant correlation was found between hepatic expression of RANKL and stage in PBC. Indeed, patients with advanced PBC demonstrated a significantly higher expression of RANKL (**Fig 4A**), whereas expression of RANK and OPG were not associated with stage in PBC (**Fig 4B and 4C**). Since OPG is the decoy receptor of RANKL, we calculated the hepatic RANKL/OPG ratio, which was found to be significantly higher in late stage PBC compared with early stage (p<0.001) (**Fig 4D**).

### Identification of Cellular Sources of RANKL/RANK/OPG in PBC

**Fig 5:**
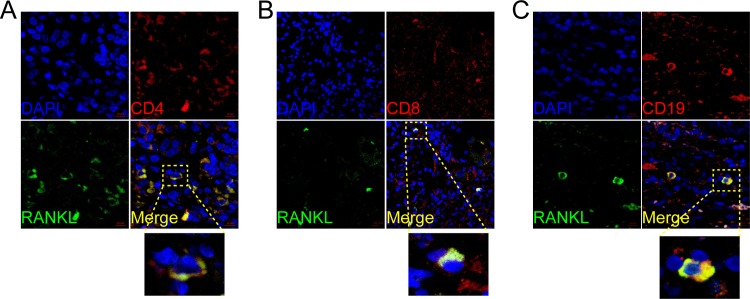
Cellular localization of the hepatic receptor activator of nuclear factor κB ligand (RANKL) in PBC. RANKL (A, B, C; green) expression in liver was analyzed by confocal scanning microscopy to identify cell types secreting the molecule. Anti-CD4 (CD4+ T lymphocyte), anti-CD8 (CD8+ T lymphocyte), anti-CD19 (B lymphocyte) are shown in green. Co-localization staining is represented by yellow (green+ red). Cell nuclei are stained in blue. Scale bar indicate 10μm.

**Fig 6:**
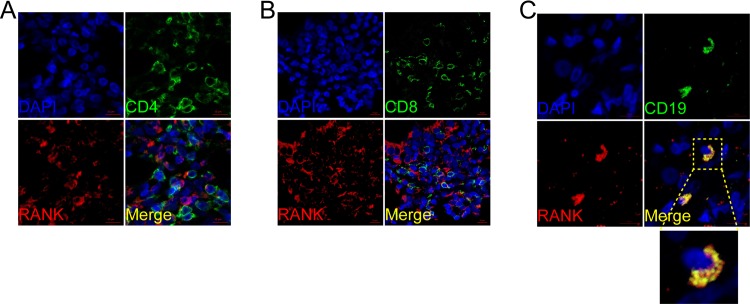
Cellular localization of the hepatic receptor activator of nuclear factor κB (RANK) in PBC. RANK (A, B, C; red) expression in liver was analyzed by confocal scanning microscopy to identify cell types expressing the molecule. Anti-CD4 (CD4+ T lymphocyte), anti-CD8 (CD8+ T lymphocyte), anti-CD19 (B lymphocyte) are shown in green. Co-localization staining between anti-RANK and anti-CD19 is represented by yellow (green+ red). Cell nuclei are stained in blue. Scale bar indicate 10μm.

**Fig 7:**
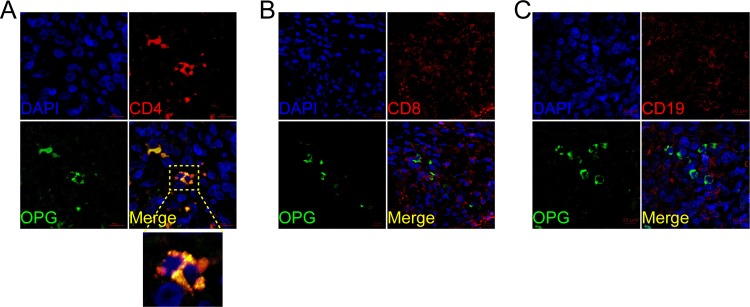
Cellular localization of hepatic osteoprotegerin (OPG) in PBC. OPG (A, B, C; green) expression in liver was analyzed by confocal scanning microscopy to identify cell types expressing the molecule. Anti-CD4 (CD4+ T lymphocyte), anti-CD8 (CD8+ T lymphocyte), anti-CD19 (B lymphocyte) are shown in red. Co-localization staining between anti-RANK and anti-CD4 is represented by yellow (green+ red). Cell nuclei are stained in blue. Scale bar indicate 10μm.

We also performed immunofluorescence double staining for CD4, CD8, CD19, CD11c, CD56 and CD68 to identify the cell type(s) expressing RANK, RANKL, and OPG. In PBC, RANKL predominantly co-localized with CD4+, CD8+ and CD19, suggesting that T and B lymphocytes were the primary sources (**Fig 5**). In contrast, RANK co-localized with CD19 but was not detected on CD4+ or CD8+ T lymphocytes (**Fig 6**). CD56 and CD68 positive cells were RANK positive **(S1 Fig)**. OPG was expressed by CD4+ T lymphocytes but not the CD8+ T lymphocytes or B lymphocytes (**Fig 7**). Lymphocyte subsets were also studied in samples from AIH, PSC, and CHB patients, as well as HC, and were found to be not specific, and not related to the inflammation and fibrosis grade (data not shown).

### RANK Is Expressed by Damaged Cholangiocytes in PBC

**Fig 8:**
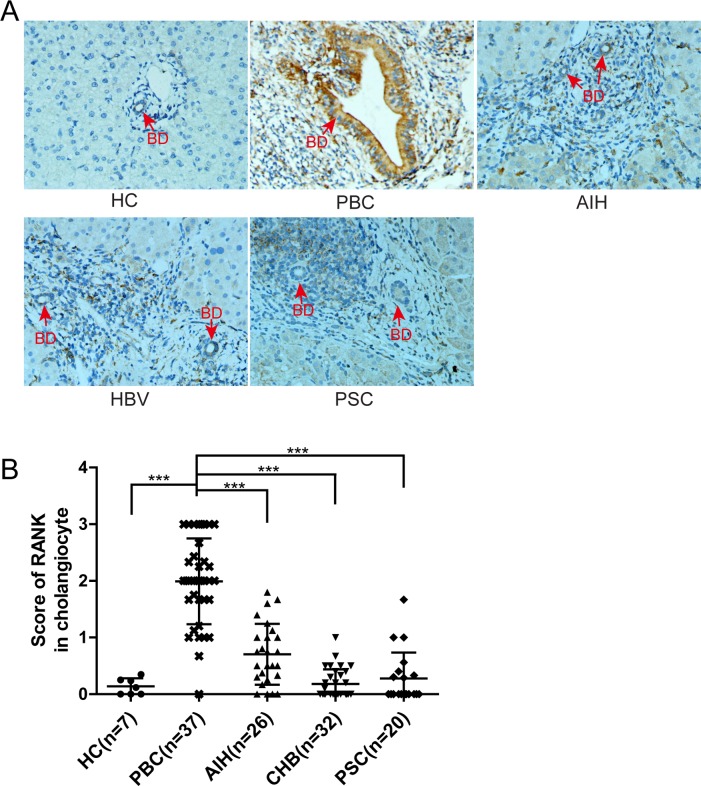
RANK expressed in cholangiocytes was elevated in patients with PBC. (A) Representative staining images (magnification 400×) from the portal areas in PBC, AIH, CHB, PSC and unaffected controls are shown (BD, bile duct, red arrow); (B) Comparison of cholangiocytic RANK levels in PBC versus unaffected and other chronic liver disease controls. Red arrows indicate bile ducts. (***, P<0.001).

The immunohistology staining demonstrated that RANK was also expressed in biliary epithelial cells, especially the degenerated cholangiocytes of interlobular bile ducts in PBC (**Fig 8A**). To determine whether RANK(+)ve cholangiocytes were specific for PBC, we determined the level of RANK expression in cholangiocytes from PBC, unaffected controls and chronic liver diseases. Importantly, RANK expression in cholangiocytes was significantly higher in PBC than AIH, CHB or PSC (**Fig 8B**, p<0.001).

## Discussion

There has been an enormous effort in both human and murine systems to identify factors that lead to the development of PBC, both on the cellular and molecular basis [3, 27–34]. Such efforts have not translated into novel therapy or the use of biologics in PBC [35–40]. Our data reflects the hepatic expression and cellular localization of RANKL/RANK/OPG in PBC. We report that the expression of RANKL/RANK/OPG were all markedly elevated in the liver of PBC compared to extensive use of controls. Most importantly, the hepatic level of RANKL was associated with disease severity. There is limited information previously published on the role of RANK/RANKL in PBC; most previous data focused only on bone metabolism. Previous studies have suggested that a dysregulated innate immune response is mediated by members of the TNF superfamily and downstream inflammatory signaling pathways in PBC [41, 42]. Recently novel members of this superfamily, including the RANKL/ RANK/ OPG molecules, have been found to be a link between inflammatory cells and autoimmunity. Both *in vitro* and *in vivo* studies have indicated that RANKL/RANK interaction activates the immune response by promoting the survival of DCs [43, 44]. Moreover, the intricate RANKL/RANK/OPG signal pathways potentially mediates self-tolerance and oral-tolerance through regulatory T cell functions [45]. Of particular interest, accumulating evidence suggests the importance of RANKL signaling in the prevention and suppression of autoimmunity. Indeed, studies in murine models reflect that deficiencies in RANKL, RANK or their downstream molecules in the signal pathway is sufficient to induce autoimmunity, resulting in severe disease phenotypes [46–49]. Activation of RANKL/RANK/OPG system has been implicated in rheumatoid arthritis (RA), Crohn’s disease (CD) and ulcerative colitis (UC) [16, 50]; moreover RANKL blockade improves hepatic insulin resistance and prevents development of diabetes [51].

SNP analysis has revealed that the RANKL gene is near a novel risk locus for PBC, suggesting a role of RANKL/RANK/OPG system in the pathogenesis of PBC. PBC patients had higher serum OPG levels but lower serum RANKL levels compared to unaffected controls [52]. Our data however demonstrate a high expression of RANKL in liver, suggesting that the level of RANKL in circulation does not reflect the actual level of this protein in the disease site of PBC. As earlier reported, higher serum OPG levels were associated with advanced PBC disease, whereas lower serum RANKL level was associated with low osteocalcin level. However, no significant differences in hepatic OPG mRNA level were found between PBC and controls [53]. This latter study included only a relatively small sample size. We should emphasize that it is known that post transcriptional events play an important role in the overall expression of RANKL/RANK/OPG proteins [54].

Our data also indicate that RANKL is expressed predominantly by CD4+ and CD8+ T lymphocytes and CD19+ B lymphocytes, whereas B lymphocytes expresses RANK, the cognate receptor for RANKL. One of the most important findings in our study is that RANK is also expressed by cholangiocytes. As cholangiocytes are the major victims in PBC, RANKL-RANK interaction may directly affect the pathogenesis of cholangiopathy in PBC. OPG, the decoy receptor for RANKL, was primarily expressed by CD4+ T lymphocytes.

**Fig 9:**
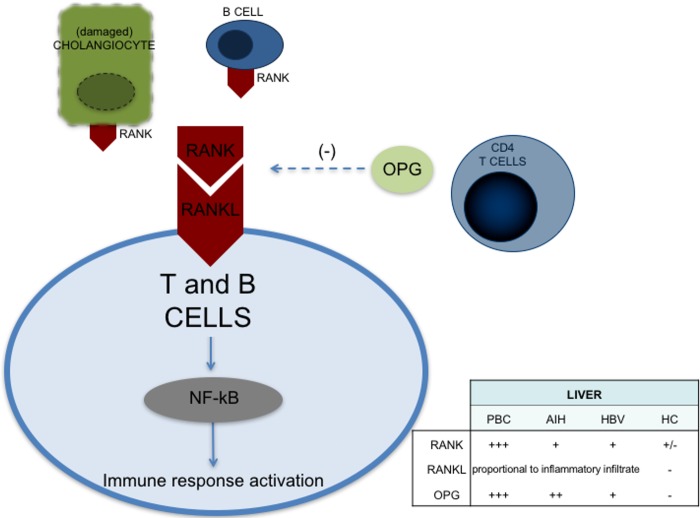
Graphic representation of the cell types expressing RANK/RANKL/OPG in PBC.

Little is known regarding the role of RANK and RANKL in lymphoid subpopulations. Tumor-infiltrating regulatory T cells have been reported to stimulate mammary cancer metastasis through RANKL-RANK signalling [55]; and RANKL-positive expression colocalized with CD4+ and CD8+ T lymphocytes in acutely rejected kidney tissue in an animal model of kidney transplantation [56]. To our knowledge, deficiencies in RANK/RANKL have not been investigated in PBC murine models. Based on our results, we postulate that the RANK/RANKL axis in PBC potentiates the inflammatory response against cholangiocytes. Indeed, damaged cholangiocytes in PBC express high levels of RANK which leads to the recruitment of RANKL positive T cells and exacerbates the immune attack against biliary cells. On the other hand, the presence of RANKL would recruit OPG, which could sequester the immunoregulatory activity of RANKL and promote the infiltration of inflammatory cells in the portal tract (Fig 9).

Our study has some weaknesses that should be discussed. First, the number of unaffected subjects included is limited. These extremely difficult to obtain samples derive from subjects undergoing both liver surgery for benign lesions and liver donors. In order to address the the lack of unaffected subjects in our study we included a sufficient number of subject with non-cholestatic liver disease (CHB). Second, functional data are missing; indeed, further studies should investigate RANKL/RANK/OPG signaling in PBC murine model.

In conclusion, our findings provide the first evidence for an active RANKL/RANK/OPG signaling in PBC, a potential target of immunotherapy. If so, translation to the clinic may be feasible given current pharmacological strategies to lower RANKL activity to treat osteoporosis.

## Supporting Information

S1 Fig Cellular localization of hepatic RANK in PBC. RANK (red) expression in liver was analyzed by confocal scanning microscopy to identify cell types expressing the molecule. Anti-anti-CD11c, anti-CD56, and anti-CD68 are shown in green. Co-localization staining represented by yellow (green+ red). Cell nuclei are stained in blue (DAPI). Scale bar indicate 10μm.
(TIFF)
