# Supplementary figures and images for: Quantitation of the Rank-Rankl Axis in Primary Biliary Cholangitis
Source: PLoS One. 2016 Sep 15;11(9):e0159612. doi: 10.1371/journal.pone.0159612 (PMC5025177; doi:10.1371/journal.pone.0159612)

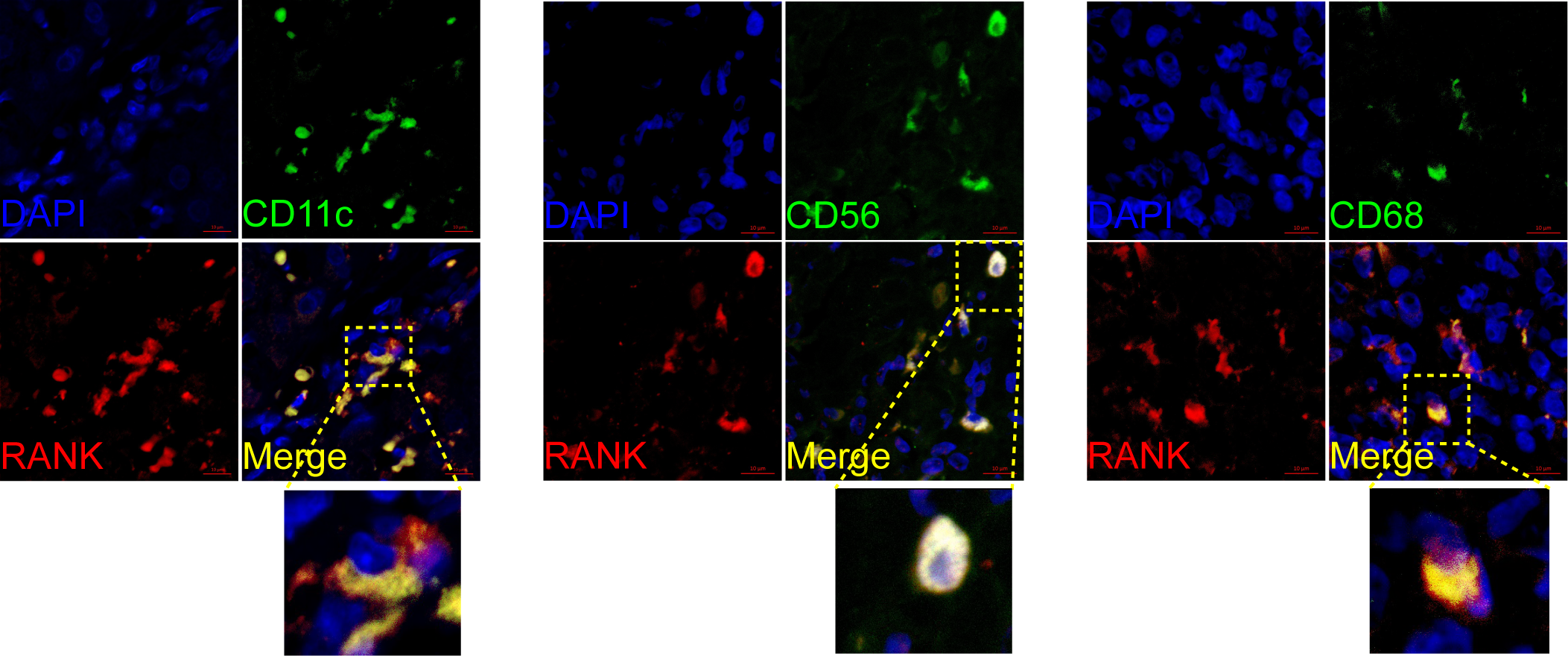

Supplement: S1 Fig — RANK (red) expression in liver was analyzed by confocal scanning microscopy to identify cell types expressing the molecule. Anti-anti-CD11c, anti-CD56, and anti-CD68 are shown in green. Co-localization staining represented by yellow (green+ red). Cell nuclei are stained in blue (DAPI). Scale bar indicate 10μm. (TIFF) [file pone.0159612.s001.tiff]
